# Prevalence of shiga toxin producing *Escherichia coli*, *Salmonella enterica*, and *Listeria monocytogenes* at public access watershed sites in a California Central Coast agricultural region

**DOI:** 10.3389/fcimb.2014.00030

**Published:** 2014-03-04

**Authors:** Michael B. Cooley, Beatriz Quiñones, David Oryang, Robert E. Mandrell, Lisa Gorski

**Affiliations:** ^1^Produce Safety and Microbiology Research Unit, Agricultural Research Service, US Department of AgricultureAlbany, CA, USA; ^2^Division of Risk Analysis, Center for Food Safety and Applied Nutrition, Food and Drug AdministrationCollege Park, MD, USA

**Keywords:** STEC O157:57, STEC non-O157, *Salmonella*, *Listeria monocytogenes*, watersheds, agriculture, leafy-vegetable production, prevalence

## Abstract

Produce contaminated with enteric pathogens is a major source of foodborne illness in the United States. Lakes, streams, rivers, and ponds were sampled with Moore swabs bi-monthly for over 2 years at 30 locations in the vicinity of a leafy green growing region on the Central California Coast and screened for Shiga toxin producing *Escherichia coli* (STEC), *Salmonella enterica*, and *Listeria monocytogenes* to evaluate the prevalence and persistence of pathogen subtypes. The prevalence of STEC from 1386 samples was 11%; 110 samples (8%) contained *E. coli* O157:H7 with the highest prevalence occurring close to cattle operations. Non-O157 STEC isolates represented major clinical O-types and 57% contained both shiga toxin types 1 and 2 and intimin. Multiple Locus Variable Number Tandem Repeat Analysis of STEC isolates indicated prevalent strains during the period of study. Notably, *Salmonella* was present at high levels throughout the sampling region with 65% prevalence in 1405 samples resulting in 996 isolates with slightly lower prevalence in late autumn. There were 2, 8, and 14 sites that were *Salmonella*-positive over 90, 80, and 70% of the time, respectively. The serotypes identified most often were 6,8:d:-, Typhimurium, and Give. Interestingly, analysis by Pulsed Field Gel Electrophoresis indicated persistence and transport of pulsotypes in the region over several years. In this original study of *L. monocytogenes* in the region prevalence was 43% of 1405 samples resulting in 635 individual isolates. Over 85% of the isolates belonged to serotype 4b with serotypes 1/2a, 1/2b, 3a, 4d with 4e representing the rest, and there were 12 and 2 sites that were positive over 50 and 80% of the time, respectively. Although surface water is not directly used for irrigation in this region, transport to the produce can occur by other means. This environmental survey assesses initial contamination levels toward an understanding of transport leading to produce recalls or outbreaks.

## Introduction

Consumption of contaminated produce accounts for nearly half of the outbreak-associated foodborne illnesses in the United States, and leafy vegetables are associated with more illness than any other produce commodity (Gould et al., 2013; Painter et al., 2013). Vegetables are a common cause of foodborne illness in the European Union as well (EFSA and ECDPC, 2012). Produce can become contaminated at any point in the production chain as long as a source of the pathogen is present in the vicinity. Public waterways such as rivers, lakes, ponds, and streams can provide a central reservoir for pathogen contamination (Hanning et al., 2009; Lynch et al., 2009; Oliveira et al., 2011). The water can become contaminated from a variety of sources such as exposure to wildlife, sewage, and agricultural runoff from animal operations (Gagliardi and Karns, 2000; Walters et al., 2011). Wildlife can become contaminated also through exposure to contaminated water with subsequent deposit of pathogens onto fields (Fenlon, 1985; Kirk et al., 2002; Jay et al., 2007; Gorski et al., 2013). Furthermore, these natural waterways can flood after large rain events leading to pathogen transmission into the fields (Cooley et al., 2007).

Most cases of bacterial foodborne illness and recalls linked to contaminated produce are associated with *Escherichia coli* and *Salmonella enterica* (CDC, 2008; FDA, 2009, 2010b,c) Also, in recent years there have been several high profile outbreaks associated with *Listeria monocytogenes* contamination of produce and the number of produce recalls due to *L. monocytogenes* are also increasing (Doell, 2010; FDA, 2010a,d, 2012; CDC, 2013). We initiated a survey of several public watersheds in a major leafy green production region of Central Coastal California to determine the prevalence of shiga toxin (*stx*) producing *E. coli* (STEC) including *E. coli* O157:H7, *Salmonella*, and *L. monocytogenes*. The watershed is never used for field irrigation directly, but is available to wildlife, and serves as an indicator of the levels of these foodborne pathogens in the environment.

This survey was part of a project to obtain pathogen prevalence data that will facilitate improved spatial and temporal resolution of prevalence by parameterizing and validating a Predictive Geospatial Risk Assessment Model (PGRAM) developed by the Food and Drug Administration in collaboration with the National Aeronautics and Space Administration (NASA). Similar models have been used to successfully predict Rift Valley fever and other zoonotic pathogens with ecological drivers using geospatial climate data (Anyamba et al., 2009; Moore et al., 2012; Semenza et al., 2013). The present report reveals 2 years of pathogen prevalence data for the 3 foodborne pathogens in this ongoing survey of 30 different sites, representing 5 watersheds in a major produce production region that were sampled on average twice a month beginning in October, 2011.

## Materials and methods

### Sampling

**Figure 1:**
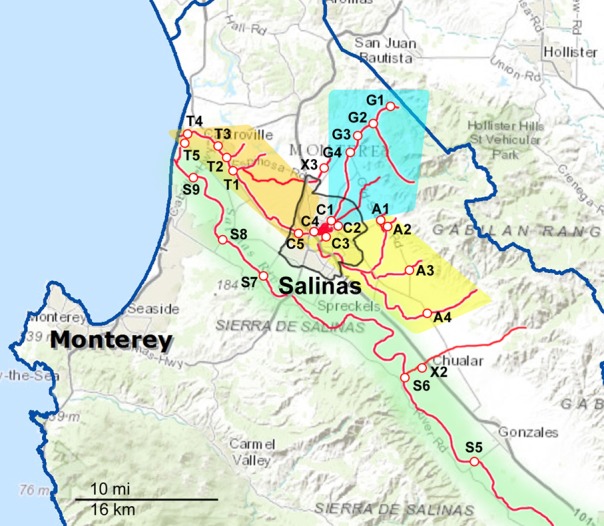
**Maps of the Northern part of the sampling areas and watersheds.** The waterways are marked as red lines, and the sampling sites are indicated along the red lines. The sampling sites are labeled with a letter corresponding to the watershed to which they have been assigned and a number to differentiate between sites within that watershed. A, Alisal Creek; C, Carr Lake; G, Gabilan Creek; S, Salinas River; T, Tembladero Slough. X indicates sites that were sampled regularly but do not fit into any of the designated watersheds.

**Figure 2:**
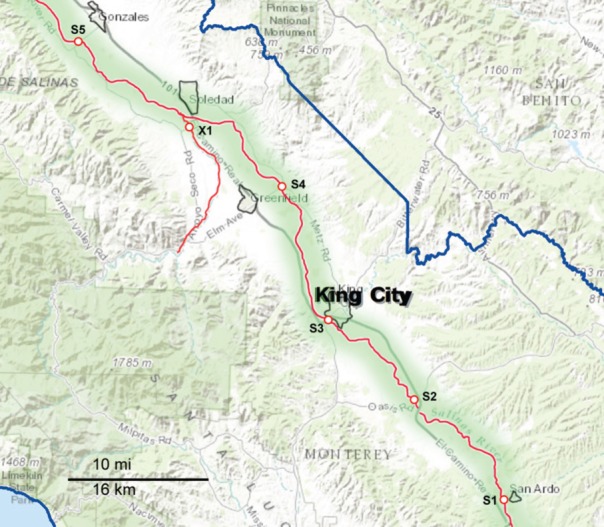
**Map of the Southern part of the sampling area, consisting primarily of the upper Salinas River (S1 through S5).** The waterways are marked as red lines, and the sampling sites are indicated along the red lines. The sampling sites are labeled with a letter corresponding to the watershed to which they have been assigned and a number to differentiate between sites within that watershed. X indicates sites that were sampled regularly but do not fit into any of the designated watersheds.

Sampling sites in Monterey County were selected based on both accessibility and previous sampling expertise gained from a collaboration with the Central Coast Water Quality Control Board. Results from this collaborative survey have been published and served as the initial data for population of the PGRAM model (Cooley et al., 2007). Consequently, many of the initial sampling sites were incorporated into the present survey. Moore swabs (cut cotton gauze tied to a string) were deployed in lakes, rivers, streams, and ponds in Monterey County for up to 24 h as described previously (Barrett et al., 1980). Water sampling locations are shown in Figures 1, 2. In addition to water samples, during the spring and summer months, fecal samples under swallow nests at the same locations along the Salinas River were collected in sterile 50 mL conical tubes. Most sites were grouped into watersheds. Sampling occurred usually every 2 weeks over a 2 year period (October 2011–November 2013). Not all sites were sampled on each sample date due to lack of water (site may have dried) or loss of the swab (swab was gone upon return to the site). Additional sample dates were included to take advantage of rain events since previous results indicated higher prevalence of *E. coli* O157:H7 during these times (Cooley et al., 2007). Swabs were placed into 3.6 L WhirlPak bags, kept on ice, transported to the lab, and processed immediately. Five hundred microliters of sterile water was added to each bag followed by vigorous shaking by hand for 20 s. One hundred microliters was removed for *L. monocytogenes* isolation (see below). One hundred and fifty microliters was used for other research not described here. The remainder of the sample (including the swab and all liquid/debris) was transferred to a 24 oz. stand-up Whirlpak bag and 30 ml of 10× Tryptic Soy Broth (TSB) was added. For bird fecal samples, 10 g were added to 100 mL of TSB. Incubation of both swab and feces was at 25°C for 2 h, then 42°C for 8 h, and the samples were held at 4°C until the following morning. The TSB enrichment cultures were used for STEC *E. coli* and *Salmonella* isolations. Also, 1 mL of the enrichment was frozen in 1 M glycerol and stored at −80°C for potential subsequent analyses.

### STEC O157 and non-O157 STEC isolation

STEC O157 and other STEC isolation was by methods published previously (Cooley et al., 2013). Briefly, 1 mL of TSB enrichment was centrifuged and the pellet resuspended in the same volume of sterile water. Genomic DNA was released from 100 uL with a boil preparation and 5 μL was used as a template for real-time PCR (RT-PCR) amplified using multiplex primers designed to detect all *stx* types (Cooley et al., 2013). Enrichments which produced Cycle Threshold (Ct) values below 27 were considered potentially positive for STEC. These enrichments were streaked onto CHROMagar O157 media (DRG International, Mountainside, NJ, USA) and single mauve, *E. coli*-like colonies were selected for RT-PCR amplification using the *stx* multiplex primers. Colonies with Ct values less than 20 were stored for further characterization.

In a parallel procedure, 1 mL of each enrichment was subjected to Immuno Magnetic Separation (IMS) with 20 μ L of magnetic beads conjugated with anti-O157 antibody (Invitrogen/Dynal, Carlsbad, CA) using the Dynal BeadRetriever (Invitrogen/Dynal, Carlsbad, CA) and the EPEC/VTEC protocol established by the manufacturer. Fifty microliters each of resuspended IMS beads were spread on 3 media: CT-SMAC, Sorbitol MacConkey agar (Difco Labs; Detroit, MI) containing cefixime (0.05 μ g/mL; Invitrogen/Dynal) and tellurite (2.5 μ g/mL; Invitrogen/Dynal); NT-RA, Rainbow Agar O157 (Biolog, Hayward, CA) containing novobiocin (20 μ g/mL; Sigma-Aldrich) and tellurite (0.8 μ g/mL; Invitrogen/Dynal); and mSBA, modified Sheep Blood Agar with mitomycin C (Sigma-Aldrich, St. Louis, MO), and X-Gal (Teknova, Hollister, CA), as described previously (Cooley et al., 2013). All plates were incubated at 37°C for 24 h. Suspect O157:H7 colonies were selected based on colony color and were subjected to RT-PCR for the presence of the O157 O-antigen synthesis (*rfb*E) and intimin (*eae*) genes (Cooley et al., 2007). Additionally, non-O157 *E. coli*-like colonies were selected from NT-RA (red colonies) and mSBA (blue colonies that showed hemolysis activity) and analyzed using the multiplex RT-PCR procedure for the presence of the *stx* genes described above.

### Genotyping of STEC O157 and STEC isolates

STEC O157 strains were analyzed by an 11-loci MLVA method described previously (Cooley et al., 2010). Non-O157 STEC strains were analyzed by the 10-loci MLVA method which includes 7 loci initially described for *E. coli* (Lindstedt et al., 2007; Cooley et al., 2013) and 3 additional loci including a CRISPR locus (Løbersli et al., 2012). Briefly, overnight cultures were boiled and aliquots were used as template in multiplex PCR reactions with fluorescently labeled primers to amplify fragments containing tandem repeats. Amplified fragments were size-fractionated by an ABI 3130 sequencer (Applied Biosystems, Foster City, CA), the data imported into Bionumerics software 6.01 (Applied Maths, Austin, TX). and allele numbers were assigned by Multi-Locus Variable Repeat Tandem Repeat Analysis (MLVA) algorithms as described (Cooley et al., 2013). STEC genotyping was supplemented with sequencing of 350 bp in the hypervariable region of the *ompA* gene as described (Cooley et al., 2013). Sequence alignments and allele assignments were produced from dendrograms within BioNumerics (Applied Maths, Belgium). Additionally, STEC, MLVA and *ompA* data were analyzed together to create a single genotype by creation of a composite similarity matrix as described previously (Cooley et al., 2013). STEC strains were also serotyped by PCR for O-type using sequence-specific primers targeting O-antigen gene clusters, as previously described (Quiñones et al., 2012).

### *salmonella* enrichment, isolation, and confirmation

The same TSB cultures used for *E. coli* isolations were used for *Salmonella* culturing in two parallel procedures, as was done previously (Cooley et al., 2007; Kalchayanand et al., 2009; Gorski et al., 2011b, 2013).Briefly in one method the TSB enrichments were plated onto Modified Semi-solid Rappaport Vasilliadis (MSRV) medium and resulting motile growth was picked and streaked onto Xylose Desoxycholate Agar (XLD). In the parallel method aliquots of the TSB cultures were incubated for 10–30 min at room temperature with anti-*Salmonella* IMS beads (Dynal, Invitrogen), and the beads collected with an automatic bead retriever (Invitrogen) according to manufacturer's instructions. The beads were then inoculated into Rappaport-Vasilliadis Soya Peptone Broth (RVS, Oxoid, Remel, Inc., Lenexa, KS), which was incubated at 42°C and the resulting growth was streaked onto XLD. All XLD plates were incubated at 37°C. Up to 5 black colonies were picked from the XLD plates from both methods and confirmed as *Salmonella* via PCR directed against *invA* (Gorski et al., 2011b, 2013).

To determine if colonies selected from a sample were siblings, they were screened with repetitive-element PCR (rep-PCR) with ERIC-1R and ERIC-2 primers (Albufera et al., 2009), but with modifications specified previously (Gorski et al., 2013). Rep-PCR patterns were compared, and colonies representing each pattern for a given sample were selected for Pulsed-field gel electrophoresis (PFGE) analysis with *Xba*I (Ribot et al., 2006). Images of PFGE patterns were imported into BioNumerics, and bands >33 kb assigned visually using band assignment. A standard isolate (*S.* Braenderup H9812) was run on each gel to allow comparisons between gels. PFGE patterns that were direct matches to isolates previously serotyped were assigned those serotypes. Isolates with unique patterns were sent to the USDA-APHIS National Veterinary Services Laboratory (Ames, IA) for serotyping. Profile similarities were calculated using Dice binary coefficients with optimization set to 1.5%, and band position tolerances of 1.5%. Cluster analysis was done using the unweighted pair group method with arithmetic averages (UPGMA). Uncertain bands were excluded from analysis.

### *L. monocytogenes* enrichment, isolation, confirmation, and serotyping

Twenty-five microliters of 5X Buffered *Listeria* Enrichment Broth Base (BLEB, Difco, Becton-Dickinson-BBL, Franklin Lakes, NJ) were added to 100 ml of Moore swab rinsate (described above) in 500 ml Whirlpak bags, and the bags were incubated on a rotating shaker at 150 rpm at 30°C for 18 h, and then held at 4°C until the following morning. Anti-*Listeria* IMS beads (Invitrogen, Dynal) were added to the enrichment cultures (20 ul of beads into 1 ml of culture) and the cell-bead suspensions were incubated, rinsed, and collected in a bead retriever using manufacturer's instructions (Invitrogen, Dynal,). Beads were collected into 130 ul of Phosphate Buffered Saline with Tween (PBS-Tween: 10 mM sodium phosphate, pH 7.2, 150 mM NaCl, 0.05% Tween-20). The beads were used in two parallel methods to isolate *L. monocytogenes*.

Upon collection of the beads, 30 ul were deposited onto a Brilliance *Listeria* Agar plate (Oxoid, Remel, Lenexa, KS) and streaked to obtain isolated colonies. The remaining 100 μl of bead suspension were inoculated into 5 ml of Fraser broth (Difco) supplemented with ferric ammonium citrate (0.5 g/L), and incubated at 37°C for 2 days. Thirty microliters of black Fraser broth cultures were inoculated onto Brilliance *Listeria* agar plates, and streaked for isolated colonies. All Brilliance plates were incubated for 2 days at 37°C. Up to three colonies that were blue with a zone of clearing on Brilliance plates were picked, deposited onto Trypticase Soy Agar plates (TSA, Difco), and grown overnight at 37°C. Isolates were further cleaned by a passage on Modified Oxford Agar (MOX, Difco), which was incubated at 37°C. Isolated bluish-white colonies with a zone of black precipitate on MOX were streaked onto TSA for further analysis. Hemolysis was tested by depositing isolates onto TSA containing 5% sheeps blood (Remel), and incubating up to 5 days at 37°C. Clearing underneath colonies could be discerned by holding the plates up to a light to determine transparency.

Isolates were subjected to PCR for the *hlyA* gene using the primers and protocol of Norton et al. (2001), using One*Taq* Hot Start (New England Biolabs, Ipswich, MA), and the PCR products were visualized on a 1% agarose gel stained with GelRed (Phenix Research Products, Candler, NC). PCR template was made by picking a piece of colony with a sterile, disposable needle and depositing it into a PCR tube that contained 50 μl of 1X PCR buffer and 50 ul of 1% Triton X-100. The tubes were incubated in a thermocycler for 15 min at 100°C. After the tubes had cooled, 5 μl of this cell suspension was used in PCR reactions. Isolates yielding a 858 bp amplicon were considered positive tentatively and were serotyped for O-antigen by an ELISA serotyping method (Palumbo et al., 2003). Any isolate that was not clearly designated as serotype 1/2, 3, or 4 by O-typing sera were discarded from further analysis. ELISA serotyping using O-antigen antisera is sufficient to determine any serotype 4 isolate. Isolates that were O-serotype 1/2 or 3 were further subtyped for H-antigen using the multiplex PCR serotyping method of Doumith et al. (2004).

### Statistical analysis

Comparisons between sample treatments or correlations of prevalence values and comparisons of prevalence between seasons were analyzed by One Way ANOVA or Kruskal–Wallis ANOVA using Sigma Stat version 3.0 (SPSS, Chicago, IL) or GraphPad Prism 6.03 (GraphPad Software, La Jolla, CA). Five day precipitation summation were computed from four weather sites in Monterey County from the California Weather Database (http://www.ipm.ucdavis.edu/) and designated as SALINAS, NSALINAS, GONZALAS, CASTROVL.

## Results

### Sampling and watershed groupings

Sampling occurred biweekly during the 2 year period resulting in 1405 samples processed. Differences in the prevalence of pathogens were noted for some of the organisms between sample dates and specific locations. However, the small number of samples per location and sampling trip was often too small to allow statistical analysis. These location and seasonal differences were clearer if grouped by watershed. The sampling sites were grouped into five watersheds as indicated in Figures 1, 2. The Salinas River (9 sampling sites) is the central waterway in the region, and it flows northwest into the Pacific Ocean. The Alisal and Gabilan watersheds (each of which had 5 sampling sites) empty into Carr Lake (6 sampling sites) and eventually into the Tembladero Slough (5 sampling sites). In addition to the sites located in watersheds, there were 3 sites that could not be placed into any watershed. However, the data from those 3 independent sites are included in the overall prevalence data. The winter season was defined as starting on December 1 to match more closely the normally expected rain fall totals in the region.

### STEC prevalence

Overall prevalence at all sampling sites for the entire sample period was 8% for STEC O157 and 11% for non-O157 STEC (Table 1). However, the prevalence in individual watersheds varied considerably compared to initial results. Two “hot spots” were evident. Samples from the Gabilan were 22 and 19% positive for STEC O157 and non-O157 STEC, respectively. Samples from the entire Salinas River were 16% positive for non-O157 STEC, yet significantly more non-O157 STEC samples were positive from the upper compared to the lower Salinas River (23 vs. 6%, respectively). In contrast, STEC O157 positive samples were more uniformly distributed in samples from the Salinas River.

**Table 1:** **Prevalence of STEC O157 and non-O157 STEC in watersheds**.

**Watershed**	**Prevalence ± *SD*^1^**	
	**O157**	**Non-O157 STEC**
All	8 ± 13%	11 ± 7%
Gabilan	22 ± 11%a^2^	19 ± 5%a
Salinas River	5 ± 5%b	16 ± 10%a
Upper Salinas	7 ± 5%b	23 ± 6%a
Lower Salinas	2 ± 2%b	6 ± 5%b
Tembladero	9 ± 7%b	3 ± 3%b
Alisal Creek	7 ± 8%b	22 ± 14%a
Carr Lake	6 ± 3%b	5 ± 3%b
*P*	0.005	0.019

1 SD, standard deviation.

2 Lower case letters indicate statistical groupings within a column (for the different watersheds only). Shared letters indicate no statistical difference.

The entire sample set demonstrated a seasonal effect with O157 but not with non-O157 STEC (Table 2). However, there were differences within the individual watersheds. The prevalence of both non-O157 STEC and O157 strains was highest in samples from the Gabilan watershed, ranging from 12 or 13% during the summer and fall, to 26 or 22% during the winter and spring months for STEC O157 and non-O157 STEC, respectively. Likewise, the prevalence of O157 increased significantly at the Alisal and Carr Lake sites, but was insensitive to season on the Salinas River and the Tembladero Slough. The prevalence of non-O157 STEC strains increased significantly from 12 to 22%, respectively, during the summer and fall months in samples from the Salinas River. The prevalence of both STEC O157 and STEC in samples from the Gabilan was significantly different from samples from Carr Lake at all seasons of the year. Likewise, persistence in samples from the Gabilan was different from other watershed locations, but dependant on season, indicating a consistent “hot spot” in the Gabilan watershed. For instance, prevalence of both STEC O157 and non-O157 STEC was significantly higher in the Gabilan compared to the other three watersheds during the winter and spring, except for a high prevalence of non-O157 STEC in the Salinas River.

**Table 2:** **Seasonality of STEC O157 and non-O157 STEC by watershed**.

**Watershed(s)**	**STEC O157 prevalence ± *SD*^1^**		***P*^2^**	**Non-O157 STEC prevalence ± *SD***		***P*^2^**
	**Summer-Fall**	**Winter-spring**		**Summer-fall**	**Winter-spring**	
All	4.3 ± 4.1%	11 ± 14%	0.027	12 ± 7%	10 ± 7%	0.407
Gabilan	12 ± 5%a^3^	26 ± 6%a	0.032	13 ± 5%a	22 ± 4%a	0.050
Salinas River	4.2 ± 7%a	5 ± 9%b	0.904	22 ± 19%a	12 ± 16%ac	0.026
Tembladero	4.8 ± 14%a	12 ± 25%b	0.220	1.6 ± 5%b	3.5 ± 11%b	0.676
Alisal	1.1 ± 5%b	7.8 ± 16%b	0.034	16 ± 18%a	10 ± 15%bc	0.185
Carr Lake	1.5 ± 5%b	12 ± 22%b	0.029	4 ± 19%b	4.7 ± 12%b	0.64
*P*	0.009	0.013		<0.001	<0.001	

1 SD, standard deviation.

2 P-values in this column were calculated from the data in the corresponding row.

3 Lower case letters indicate statistical groupings within a column (for the different watersheds only). Shared letters indicate no statistical difference.

### Persistence and transport of clinically important *E. coli*

At least one STEC strain was isolated from 152 samples; a total of 578 STEC strains were characterized further. Of these, 330 strains (57%) contained both *stx* types 1 and 2 and intimin. Genotyping of STEC strains by MLVA (and *ompA* sequencing for non-O157 STEC) facilitated monitoring the persistence and potential transport of specific STEC stains. Table 3 shows the results for a small subset of clinically important O-type STEC stains that were isolated repeatedly. O26 strains were isolated from sites in the upper Salinas River over a period of 2 months during the spring and early summer of 2012. Interestingly, one of these isolates was from swallows inhabiting nests under bridges spanning the Salinas River at several sites. Similarly, an O103 strain (MLVA/*ompA* type 316-6) was isolated from bird feces collected near the Salinas River in the spring of 2013, and from water collected from other distant locations (Gabilan and Alisal Creeks) earlier that year and October of the previous year. These sample sites are not connected to each other hydrologically (Figure 1). Other non-O157 STEC were isolated from widely dispersed locations (e.g., O91, type 307-6 and O103, type 340-6) spanning 11–15 months.

**Table 3:** **Persistence and transport of clinically important O-types**.

**O-type**	**Genotype^1^**	**Sample date**	**Location(s)^2^**	**Precipitation (inches)^3^**
26	1068-6	5/9/12	S3,S4	0
		5/22/12	S4	0
		6/21/12	S4	0
		7/2/12^4^	S5	0
		7/19/12	S3,S5	0
91	307-6	5/9/12	G1,S6	0
		11/8/12	C2	0
		11/19/12	C4	0.25
		4/3/13	S2	0.07
103	316-6	10/10/12	S1,G2	0
		1/2/13	A3	0.32
		4/3/13	G1	0.07
		5/29/13^4^	S1	0
103	340-6	2/7/12	S1	0.02
		5/1/13	A3	0
		5/29/13	S2,G1	0
104	1091-2	11/8/12	C2	0
		11/19/12	C4	0.25
		12/3/12	C3	3.05
		1/2/13	A3	0.32
157	1062	10/26/11	G3	0
		3/17/12	G3,G4	1.82
		5/9/12	G2	0
		6/6/12	G2	0.31
		8/15/12	G1	0
		9/12/12	G2	0
		12/3/12	G3,G4,C1,C4,C5,T3,T5	3.05, 0.08(T3,T5)^5^
		1/15/13	G3,C4	0
		2/20/13	G3	0.21
		3/6/13	G3	0.3
		5/1/13	G3	0
157	1070	1/23/12	G4	1.29
		3/17/12	C4,T1,T3,T4	1.47, 1.82(C4)^5^
		3/26/12	C1,T1,T4	0.67, 0.65(C1)^5^
		4/10/12	C1,T1,T3,T4	0.52, 0.45(C1)^5^
		6/6/12	T1,T3	0.31
157	1075	1/23/12	G3	1.29
		10/23/12	T1,T3	0
		11/18/12	T3	0.55
		12/3/12	G3,G4	3.05
		2/20/13	G3,G4	0.21
		3/6/13	G3	0.3
		6/26/13	G1,G3,C1	0

1 Eleven loci MLVA genotype of STEC O157 and 10 loci MLVA of non-O157 STEC with ompA allele after the hyphen. MLVA and ompA allele numbers are assigned to specific genotypes randomly.

2 Locations are coded in accordance with Figure 1.

3 Precipitation values are an accumulation on the 5 days prior to the retrieval of the Moore swab.

4 MLVA 316-6 and 1068-6 isolates were recovered from bird feces.

5 Precipitation values are obtained from the closest weather station. In some cases, genotypes were at widely dispersed locations where the rain totals may be different. Distal locations are noted in parentheses.

Other strains (STEC O157 and O104) were isolated repeatedly from a specific watershed or, at least, connected watersheds. Importantly, several of these samples were collected after significant precipitation events in 2012 (January 23–December 3, Table 3). STEC O157 strains were isolated also on 1 day at widely dispersed, but hydrologically connected locations, some of which are more that 30 km apart (e.g., O157 MLVA type 1062 on December 3, 2012). O157 strains of the same MLVA types were isolated from the same watersheds again up to 19 months apart.

### *salmonella* prevalence, serotypes, and persistence

*Salmonella* prevalence for all sites over the entire sampling period was 65% (908 of 1405 samples positive). Prevalence was significantly higher in the spring and summer months, at 73 and 71%, respectively, (Table 4). Prevalence in the winter was lower compared to spring and summer, but this was not significant statistically. The prevalence in the fall was lower statistically than in the spring and summer months; however, this difference was observed only for the overall prevalence. There was no difference in prevalence within watersheds between seasons, nor was there a difference between watersheds within seasons. No location was 100% *Salmonella*-positive, however, a Gabilan location was positive for 96% of the samplings (site G2 in Figure 1). Sites S3 and S4 were positive for 90% of the samplings, and the A4, S2, S5, and S6 sites were positive for over 80% of the samples (Figures 1, 2).

**Table 4:** ***Salmonella* prevalence (average ± standard deviation) at sites by season and by watershed**.

**Watershed**	**Season**				***P*-value^1^**
	**Fall**	**Winter**	**Spring**	**Summer**	
All sampling sites	54 ± 10%A^3^	60 ± 20%AB	73 ± 14%B	71 ± 11%B	0.0011
Gabilan	62 ± 31%	71 ± 32%	77 ± 21%	69 ± 22%	0.8613
Carr Lake	40 ± 17%	50 ± 15%	60 ± 20%	52 ± 29%	0.4528
Salinas River	71 ± 26%	60 ± 16%	81 ± 22%	84 ± 26%	0.1365
Tembladero	36 ± 16%	61 ± 12%	58 ± 24%	60 ± 32%	0.2727
Alisal	39 ± 17%	41 ± 38%	49 ± 38%	54 ± 42%	0.8958
*P*-value^2^	0.0807	0.3121	0.1676	0.2555	NA

1 P-values in this column were calculated from the data in the corresponding row.

2 P-values in this row were calculated from the data in the corresponding column, except for the data in the row marked “All sampling sites.”

3 Letters indicate statistical groupings by row with values sharing letters having no statistical difference. Letters are shown only for values that have a P-value < 0.05.

**Figure 3:**
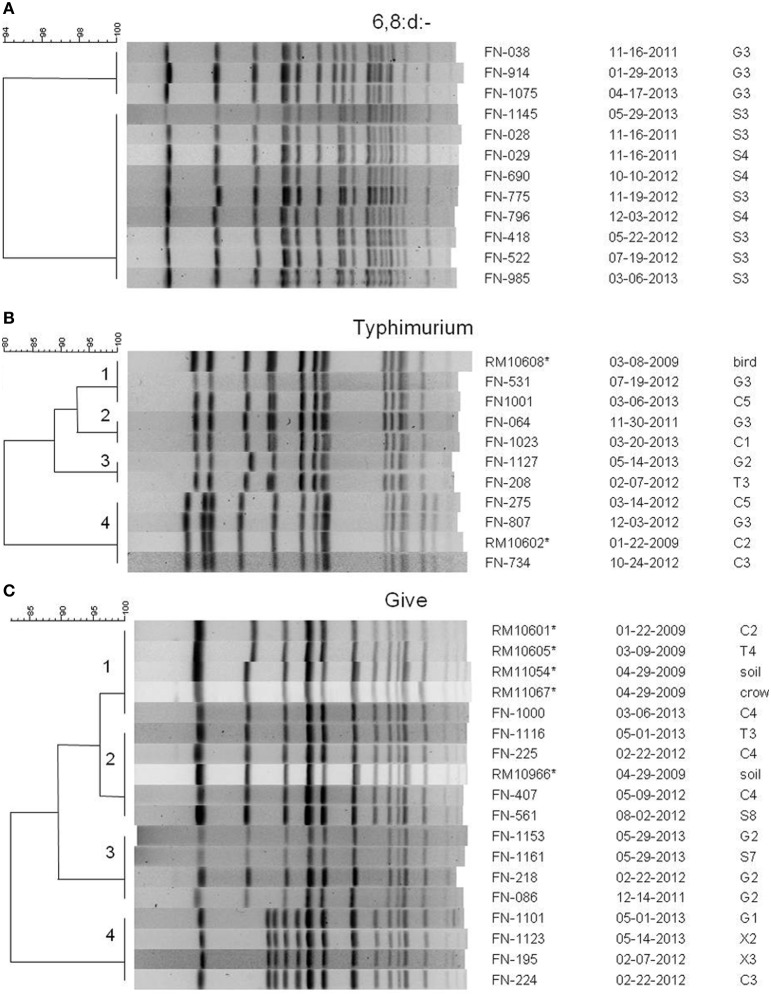
**PFGE patterns from *Xba*I digests of selected *Salmonella* isolates of serotypes (A) 6,8:d:-, (B) Typhimurium, and (C) Give.** Dendrograms were generated in BioNumerics, and the percentage similarities of the patterns are shown on each scale. Patterns are labeled with an isolate designation number, the date of sampling (month-day-year), and the site from which it was isolated. Sampling sites and designations correspond to the maps in Figures 1, 2. All isolates are from the current study except for those marked with an asterisk, which are isolates from a previous 2009 study (Gorski et al., 2011b), and are mentioned in the text.

Several potential *Salmonella* colonies were selected from each XLD plate yielding more than one type of *Salmonella* isolated from some samples. Different strains were identified using rep-PCR to limit the number of *Salmonella* isolates tested by PFGE for further characterization. There were 908 positive samples yielding 996 individual isolates selected. Over 30 different serotypes of *Salmonella* were identified; the serotypes isolated most often were the monophasic 6,8:d:-, *S*. Typhimurium, *S*. Give *S*. Oranienburg, and *S*. Montevideo. Comparisons of PFGE patterns showed persistence of some pulsotypes over time and transfer between watersheds. Serotype 6,8:d:- was isolated at least once from every watershed, and isolates representing two different *Xba*I PFGE pulsotypes continued to be isolated over 18 months from the same sites in the Gabilan and Salinas River watersheds, indicating persistence of those pulsotypes (Figure 3A).

*S*. Typhimurium isolates were isolated from each watershed, as well as, the X1, X2, and X3 sites not assigned to the watersheds (Figures 1, 2). Identical *S*. Typhimurium pulsotypes were isolated from different watersheds with some persisting for more than 3 years (Figure 3B). The pulsotype grouping “2” shown in Figure 3B represents two isolates from the Gabilan and Carr Lake watersheds that were isolated 15 months apart suggesting that this pulsotype had been transported between these connected watersheds. The pulsotype grouping “3” shown in Figure 3B also identifies a pulsotype that was transported between the connected Gabilan and Tembladero watersheds; these isolates were detected 15 months apart. The *S*. Typhimurium pulsotype groupings “1” and “4” (Figure 3B) represent isolates from the Gabilan and Carr Lake watersheds matching isolates from a previous agricultural and wildlife study of the region conducted in 2009. The *S*. Typhimurium “1” group (Figure 3B) represents the pulsotype for RM10608, a bird (spotted towhee) fecal isolate from the region in 2009 that matched two Moore swab isolates from the Gabilan and Carr Lake watersheds collected in 2012 and 2013, respectively. Strains of this pulsotype were transported apparently between wildlife and water and remained persistent in the region. The “4” grouping (Figure 3B) represented by strain RM10602 was isolated from a 2009 Carr Lake watershed sample. This pulsotype was detected again in Carr Lake and the Gabilan from samples collected in 2012 indicating persistence or reintroduction, and transfer between watersheds.

Isolates of *S*. Give were isolated from all 5 watersheds and the X2 and X1 sites shown in the maps in Figures 1, 2, respectively. Pulsotypes for selected *S*. Give isolates are shown in Figure 3C. Some of the *S*. Give pulsotypes matched those of strains from a previous 2009 survey; this result was similar to matching *S*. Typhimurium pulsotypes. The pulsotype grouping “1” shown in Figure 3C represents *S*. Give 2009 isolates from soil, a crow, and the Carr Lake and Tembladero watersheds that matched a 2013 Carr Lake isolate. The detection of this pulsotype in these locations over time indicates potential transfer between the watersheds and wildlife and the soil, as well as persistence or reintroduction after 4 years. Similarly in the pulsotype “2” grouping there is a soil isolate from the 2009 study that matched pulsotypes of isolates collected in 2012 and 2013 *S*. Give isolates from the Tembladero, Carr Lake, and Salinas River watersheds. The other two pulsotype groupings in Figure 3C show recurring pulsotypes for isolates from different watersheds detected over 18 months from samples in the present survey. Additional details regarding the characterization of *Salmonella* serotypes and the frequency in watersheds and specific locations will be reported separately.

### *L. monocytogenes* prevalence and serotypes

The prevalence of *L. monocytogenes* in the region during the period of study was 43%. The data shown in Table 5 indicates that the prevalence of the pathogen was highest in the winter and spring months (48 and 57%, respectively), and lowest in the fall at 24%. There were significant seasonal differences in *L. monocytogenes* prevalence in both the Carr Lake and Tembladero watersheds with prevalence in the winter and spring months significantly higher compared to the summer and fall months. There also were significant differences within seasons between watersheds. During the winter season the Tembladero and Salinas River watersheds had prevalences of 72 and 35%, respectively. In the summer months the Gabilan watershed had 72% positive samples for *L. monocytogenes* compared to Carr Lake and the Tembladero watersheds, which had the lowest prevalence at 24%. There was no statistical difference in the prevalence of *L. monocytogenes* in the fall and spring months between watersheds.

**Table 5:** ***L. monocytogenes* prevalence (average ± standard deviation) in the region by season and watershed**.

**Watershed**	**Season**				***P*-value^1^**
	**Fall**	**Winter**	**Spring**	**Summer**	
All	24 ± 14%A^3^	48 ± 19%BC	57 ± 17%B	34 ± 14%AC	<0.0001
Gabilan	36 ± 34%	66 ± 30%xy	74 ± 27%	72 ± 31%x	0.2222
Carr Lake	32 ± 14%A	54 ± 17%B xy	60 ± 9%B	24 ± 12%Ay	0.0003
Salinas River	17 ± 15%	35 ± 20%y	43 ± 25%	26 ± 23%y	0.0784
Tembladero	26 ± 9%A	72 ± 14%Bx	59 ± 24%B	24 ± 17%Ay	0.0005
Alisal	19 ± 11%	44 ± 15%xy	46 ± 27%	34 ± 23%xy	0.236
*P*-value^2^	0.3261	0.0155	0.1563	0.0068	

1 P-values in this column were calculated from the data in the corresponding row.

2 P-values in this row were calculated from the data in the corresponding column, except for the data in the row marked “All sampling sites.”

3 Capital letters indicate statistical groupings by row with values sharing letters having no statistical difference. Lower case letters indicate statistical groupings within a column (for the different watersheds only). Letters are shown only for values that have a p-value < 0.05.

No sampling site was 100% positive for *L. monocytogenes*; however, 96% of the samples collected at site G2 were positive. The next most positive sites were G3 (84%), X3 (67%; a small creek), and T2 (65%). These 4 sites were among the 10 that were positive for *L. monocytogenes* 50% of the time; each watershed was represented in the 10 sites.

There were 576 samples positive for *L. monocytogenes* overall; some samples yielded more than one serotype, so a total of 635 individual isolates were selected for further study. The distribution of all of the strains with their serotypes is shown in Table 6. The most common serotype was serotype 4b, which was isolated from 94% of the positive samples, and made up 85% of the isolates. Serotype 4b strains were isolated from every sampling site. Serotypes 1/2a and 1/2b each were isolated from 6% of the samples, and represented 6.7 and 5.4%, respectively, of the isolates. There were other serotypes isolated, but they were represented at a much lower frequency. Strains of serotypes 1/2a, 1/2b, and 4b were isolated during each season and over the entire sampling period, indicating that they represented strains persistent (endemic) in the watershed environment.

**Table 6:** **Numbers, serotypes and sources of *L. monocytogenes* isolates**.

**Serotype**	**Number of strains isolated**	**Watersheds and/or sites**	**Season(s)**
1/2a	43	A, C, G, S, T, X1, X3	Winter, spring, summer, fall—all years
1/2b	34	A, C, G, S, T	Winter, spring, summer, fall—all years
1/2c	2	G1, G2	Spring, winter 2012
3a	2	A, C	Winter 2013, spring 2013
4b	542	A, C, G, S, T, X1, X2, X3	Winter, spring, summer, fall—all years
4c	3	C1, G3, S4	Summer 2012, spring 2013
4d	8	A, G, S	Spring 2013, summer 2013, fall 2013
4e	1	S3	Spring 2013

## Discussion

Previous surveys of wildlife, plants, soil, water, and cattle on private lands in a Central California Coast leafy greens production region reported the prevalence of *Salmonella* and STEC for various periods of time (Cooley et al., 2007, 2013; Jay et al., 2007; Gorski et al., 2011b, 2013; Benjamin et al., 2013). The data presented in the present survey relate to samples obtained from public access water sites that represent watersheds that are central to the Salinas Valley region, a region known for producing >60% of the leafy greens for the United States between April and November. We screened for all three pathogens from the same water samples over a 2 year period, which is the first time that such a study was done in this leafy green agricultural region. Furthermore, each watershed monitored in this study is impacted uniquely by several environmental factors. Carr Lake and the Tembladero Slough are within, or downstream, of the city of Salinas, and are impacted significantly by urban runoff and seepage from septic systems. Another factor important to consider is that these two watersheds receive water continuously from the Gabilan and Alisal Creeks, especially during major rain events. Carr Lake and the Tembladero Slough are impacted continuously by runoff from agriculture, wildlife in riparian areas and runoff from cattle ranches (primarily cow/calf operations). The Salinas River, primarily due to its length, is impacted by all of the factors mentioned above. It is very important to note that the Salinas River is unusual in that it flows northward from the headwaters west of the city of Paso Robles (roughly 40 miles south of the map shown in Figure 2) and winds through eight small towns and hundreds of farms before ending at Monterey Bay near the city of Salinas and north of the city of Monterey. This region is surrounded by ranches on both sides of the valley with varying concentrations of grazing cattle. The Salinas River is bordered also by considerable riparian area throughout its length. All of these factors contribute to the novelty of this survey, which assesses the persistence and transport of pathogens in this produce production region.

The overall prevalence of STEC O157 during this 2 year study was 8% and substantially lower than previously reported for surface waters in this region from 2005 to 2006 (12%) (Cooley et al., 2007). However, the early study noted above obtained samples concentrated on the Gabilan Creek region and did not include any samples from the upper Salinas River, where the present study indicates low prevalence. Precipitation during this study was also unusually low during the winters of 2012 and 2013 compared to previous years. This has reduced runoff and wild-life and grazing activities. In contrast, the level of prevalence of non-O157 STEC levels (11%) was higher than O157 and were very similar to results from watershed samples (12%) collected during a 2.5 year study from 2008 to 2011 (Cooley et al., 2013), which may indicate different sources of contamination or that the several types of non-O157 STEC found in this region are less sensitive to seasonal variation. The current study clearly indicated that prevalence of both STEC O157 and non-O157 STEC varied substantially by watershed and pointed to an undiscovered “hot spot” in the upper portion of the Salinas River (non-O157 STEC only).

The prevalence of both STEC O157 and non-O157 STEC was different for different seasons. Previous surveys reported a correlation between rainfall and the prevalence of both O157 and non-O157 STEC (Cooley et al., 2007, 2013). However, the prevalence detected in this study did not yield statistically significant data for the typical four seasons because of the small number of samples taken compared to other studies. Nevertheless, winter and spring corresponded to elevated levels of STEC O157. In contrast, seasonality of non-O157 STEC could be demonstrated only in individual watersheds, specifically the Gabilan and the Salinas River. Interestingly, seasonality of non-O157 STEC in the Salinas River was not correlated with rainfall, being higher during the driest months in the summer and fall. We speculate that this may occur because the source of water for the Salinas River changes during the year. Rainfall during the winter and spring contributes significantly to flow in the Salinas River, but this is followed during the summer and fall months with nearly all the water in the Salinas River supplied by two reservoirs (Lake San Antonio and Lake Nacimiento) located northwest of Paso Robles. This novel observation of seasonal non-O157 STEC contamination in the Salinas River was unexpected during dry months of the year and water from these lakes and the surrounding environment will be the subject of future investigations.

Several clinically important serogroups of STEC were isolated in this survey. STEC isolated repeatedly demonstrated persistence lasting several months. How this persistence occurs is unknown. It is possible that the well-adapted strains persisted in the sediment and were re-introduced to the water stream, as has been demonstrated with coliforms and STEC O157 (Crabill et al., 1999; Czajkowska et al., 2005). A more probable prediction is the re-introduction of strains to the water from animal reservoirs, which allow for persistence at high titer for months (Sánchez et al., 2009; Döpfer et al., 2012). An important observation was the isolation of non-O157 *E. coli* from swallow feces that match water isolates found at distal locations. Swallows typically construct nests under bridges and directly over the water. Furthermore, they are known to harbor STEC and were also shown to contribute significantly to generic *E. coli* levels in the underlying waterway (Kobayashi et al., 2009; Sejkora et al., 2011). Movement of these birds and introduction of STEC into distal waterways is likely and could explain repeated isolation of non-O157 STEC at distal locations that are not connected hydrologically.

The high prevalence of *Salmonella* (64%), and its uniform presence in all the watersheds was surprising since a previous 2009 survey for *Salmonella* in the area resulted in an prevalence of 2.3% overall in water, soil, wildlife, and cattle, with a 7% prevalence in water (Gorski et al., 2011b). However, a survey of coastal waters in Central and Northern California conducted over two years in 2008 and 2009 reported that 30.7% of the samples were positive for *Salmonella* (Walters et al., 2011). It is likely the higher prevalence is due to sampling through Moore swabs, which provide not only a longer exposure to a test site compared to a single batch sample, but also the sediment captured in the swab provides a composite sample. *Salmonella* is more likely to be attached to sediment particles than as unattached single or even aggregated cells (planktonic). Canal sediment from coastal Texas waters were 47.2% positive for *Salmonella* (Goyal et al., 1977). Two methods of isolation for *Salmonella* were used in the present survey, but we speculate that the high prevalence of *Salmonella* we obtained was a result of the higher efficiency of Moore swabs in water sampling. For example, 252 water samples were tested in the 2009 survey that reported a 7% prevalence in water samples, but only 6 of those samples were taken from Moore swab (Gorski et al., 2011b). A study of North Carolina watersheds indicated a prevalence of 54% for *Salmonella*, and reported that watersheds impacted by animal agriculture, residential/industry, and forest ecosystems all supported *Salmonella* survival (Patchanee et al., 2010). Differences in residential, agricultural, and riparian impact on *Salmonella* prevalence was not differentiated in the present study.

The prevalence of *Salmonella* was lowest in the fall, increased during the winter, then was highest in spring and summer. The trend was an increase in prevalence at the onset of the winter rains, but sampling at each discrete location was insufficient to determine if the results were significant. Precipitation during the summer months is minimal and correlates with increasing temperatures. Thus, it is possible that the increasing temperatures of spring and summer contribute to a higher prevalence compared to other seasons. However, these results differ from those of a survey of coastal waterways along the Central and Northern California Coast reporting a negative correlation between *Salmonella* prevalence and higher temperatures and a positive correlation with rainfall (Walters et al., 2011). A survey for *Salmonella* in New York State measured the effect of meteorological factors on classification tree models and reported no correlation of temperature with *Salmonella* prevalence (Strawn et al., 2013). However, a survey of a rural watershed in Georgia reported that temperature or season did contribute to *Salmonella* prevalence (Haley et al., 2009). These results suggest that different environmental factors in different regions may play a role in prevalence.

*Salmonella* has been detected in animals such as mammals, reptiles, and birds, which can lead to its persistence in the environment (Smith et al., 2002; Winfield and Groisman, 2003). An important finding revealed by the subtyping of *Salmonella* strains by PFGE was that specific pulsotypes of *S*. Typhimurium and *S*. Give persisted in the study region for at least 4 years. One of the persistent *S*. Typhimurium pulsotypes (represented by the clade marked “4” in Figure 3B) matches the common PFGE pattern designated by CDC as JPXX01.0014, which was implicated in several cases of salmonellosis during a 2009 outbreak associated with an unidentified food source. The *S*. Typhimurium pulsotype “1” from Figure 3B was first detected in a spotted towhee in 2009, and was detected again in 2012 and 2013 in two different watersheds. In the case of *S*. Give there were two pulsotypes that persisted for 4 years and were isolated previously from soil or birds. These pulsotypes either persisted in the region or were reintroduced from different sources. This is consistent with *Salmonella* surviving in the environment for long periods of time, potentially in sediments and animals (Winfield and Groisman, 2003; Gorski et al., 2011b). Previous surveys of cattle (mostly cow-calf operations) in the region reported a very low prevalence of *Salmonella*, but it was detected in birds, rodents, deer, a wild pig, a coyote, snakes, and lizards (Gorski et al., 2011b, 2013). There are no other major domesticated animals raised in the area (e.g., chickens, other livestock), so these results suggest that the high prevalence of *Salmonella* in watersheds is due to cycling between wildlife and water.

*L. monocytogenes* is considered an environmental organism more than *E. coli* and *Salmonella*, because it lives in nature as a saprophyte and in association with animals. The features that allow *L. monocytogenes* to adapt to environmental conditions are just starting to be elucidated (Gray et al., 2006; Gorski et al., 2011a; McLaughlin et al., 2011; Vivant et al., 2013). Our study provides the first prevalence data for *L. monocytogenes* in the region. For comparison, a survey of surface waters in a watershed in Ontario, Canada reported an overall *L. monocytogenes* prevalence of 10% (Lyautey et al., 2007). A 2-year survey of natural and urban regions in New York reported that prevalence in surface water from natural regions was 16%, and in urban regions it was 33% (Sauders et al., 2012). Compared to these other studies it was surprising that the watersheds sampled in the present study had a high prevalence overall of 43% with seasonal variation. The highest prevalence of *L. monocytogenes* occurred during the winter and spring and corresponded to high precipitation and low air temperatures. *L. monocytogenes* can grow within a large temperature range, from refrigeration temperatures up to 42°C. It is possible that this capability facilitates *L. monocytogenes* reproduction during the colder times of the year in these niches. In a survey of agricultural regions in New York the investigators reported a higher prevalence of *L. monocytogenes* during times of cooler, above freezing temperatures (Strawn et al., 2013). This result would seem to agree with our study since the waters tested in the present study do not freeze during the winter months.

The predominance of serotype 4b isolates was surprising since other surveys of the environment and processing plants for *L. monocytogenes* isolated serotype 1/2a much more often than serotype 4b strains (Kathariou, 2002; Lyautey et al., 2007; O'Connor et al., 2010). Other surveys of watersheds for *L. monocytogenes* reported higher levels of serotype 1/2a over 4b strains (Sauders et al., 2006; Lyautey et al., 2007). The predominance of serotype 1/2a has been attributed to enhanced fitness of 1/2a strains in the environment, but an alternative explanation may be better fitness of 1/2a strains in culture media compared to other serotypes. Indeed, there is evidence for bias in some selective media in favor of serotype 1/2a over serotype 4b (Bruhn et al., 2005). The enrichment protocol described in the present study was developed specifically for this project, and the primary enrichment media (BLEB) contained no selective compounds to lessen the likelihood of bias. BLEB has been reported to have minimal bias based on serotype (Gorski et al., 2006). Because this is the first survey for *L. monocytogenes* in the region and it is not known how much wildlife, soil and vegetation contribute to the potential persistence we cannot be certain if the observed predominance of serotype 4b isolates reflect a method bias or the different ecology in this region. Studies of *L. monocytogenes* ecology report survival in the environment for several months (Welshimer, 1960, 1968). Given that *L. monocytogenes* has the ability to grow in conditions of both high and low osmolarity, adapt to acidic conditions, and live inside and outside a host these would suggest that it would be a robust organism in this region (Fenlon, 1985; Kimura, 2006).

Although the three pathogens surveyed were isolated from each watershed, the data show the highest prevalence in the Gabilan watershed (Figure 1, blue shading). This watershed was a hotspot for STEC O157 and non-O157 STECs, and one site in the Gabilan was positive consistently for *Salmonella* and *L. monocytogenes* in over 80% of the sampling trips. This watershed is more elevated than others, e.g., site G1, G2, G3, and G4 are situated at approximately 135, 100, 80, and 65 m above sea level, respectively, and cattle were observed frequently, grazing near the watershed. We speculate that the combination of point sources, roaming wildlife and high run-off during heavy rain events with elevation can result in “hot-spots” for pathogen prevalence. The other STEC hot spot was the Upper Salinas River (Figure 1, green shading), and the entire Salinas River watershed had five locations that tested positive for *Salmonella* in over 80% of the samplings. There were consistent isolations of STECs over the course of the 2 years with seasonal and site differences in prevalence and a few obvious hot spots as noted. In contrast, *L. monocytogenes*, and especially *Salmonella*, were detected at more locations consistently within the watersheds. The data indicate that certain strains of STEC and *Salmonella* persist in the region, including the Gabilan and Salinas Rivers, but the mechanism(s) whereby these pathogens enter the region and how they survive are not known. Ecological studies of the three pathogens outside of human hosts indicate there are different methods of growth and survival for each pathogen in the environment, and it is likely that each pathogen species has diverse methods to grow, survive, and persist in this region. An important goal of these environmental surveys is to determine whether and/or how these pathogens are transported from the environment surrounding produce production to contaminate produce in the field resulting in potential recalls and/or outbreaks.

### Conflict of interest statement

The authors declare that the research was conducted in the absence of any commercial or financial relationships that could be construed as a potential conflict of interest.
