# Corrigendum: Prevalence of shiga toxin producing *Escherichia coli*, *Salmonella enterica* and *Listeria monocytogenes* at public access watershed sites in a California Central Coast agricultural region

**DOI:** 10.3389/fcimb.2014.00058

**Published:** 2014-05-05

**Authors:** Lisa Gorski, Michael B. Cooley, Beatriz Quiñones, David Oryang, Robert E. Mandrell

**Affiliations:** ^1^Agricultural Research Service, United States Department of AgricultureAlbany, CA, USA; ^2^Food and Drug Administration, Division of Risk Analysis, Center for Food Safety and Applied NutritionCollege Park, MD, USA

**Keywords:** STEC, salmonella, listeria monocytogenes, watersheds, agriculture

Page 2

Lines 23–27 in the section titled “Sampling” should read:

… Five hundred milliliters of sterile water was added to each bag followed by vigorous shaking by hand for 20 s. One hundred milliliters was removed for *L. monocytogenes* isolation (see below). One hundred and fifty milliliters was used for other research not described here…

Page 3,

The first line in the section “*L. monocytogenes* enrichment, isolation, confirmation, and serotyping” should read:

Twenty-five milliliters of 5X Buffered *Listeria* Enrichment Broth…

## Conflict of interest statement

The authors declare that the research was conducted in the absence of any commercial or financial relationships that could be construed as a potential conflict of interest.

